# Supplementary material for: Heat adaptation of phage T7 under an extended genetic code
Source: Virus Evol. 2021 Dec 1;7(2):veab100. doi: 10.1093/ve/veab100 (PMC8923235; doi:10.1093/ve/veab100)
Supplement: veab100_Supp [file veab100_supp.zip › Sequencing_Analysis.zip!/Sequencing_Analysis/S3/evidence/SNP_6.html]

BRESEQ :: Evidence


| Predicted mutation | | | | | | |
| --- | --- | --- | --- | --- | --- | --- |
| evidence | seq id | position | mutation | annotation | gene | description |
| RA | NC\_001604.1 | 21,265 | A→C | intergenic (–/–) | – / – | –/– |

| Read alignment evidence... | | | | | | | | | | | |
| --- | --- | --- | --- | --- | --- | --- | --- | --- | --- | --- | --- |
|  | seq id | position | | ref | new | freq | score (cons/poly) | reads | annotation | genes | product |
| \* | NC\_001604.1 | 21,265 | 0 | A | C | 99.0% | 5348.8 / NA | 1776 | intergenic (–/–) | *–/–* | –/– |
| Reads supporting (aligned to +/- strand):  **ref** base A (13/5);  **new** base C (877/878);  **total** (891/885) | | | | | | | | | | | |
| Fisher's exact test for biased strand distribution *p*-value = 9.46e-02 | | | | | | | | | | | |
| Kolmogorov-Smirnov test that lower quality scores support variant *p*-value = 1.00e+00 | | | | | | | | | | | |

Only 100 of 1777 total aligned reads displayed.  

`ATCCTAAAGAGGCTTGCCCATACATCCCGATTCGGATGGTCAGACTAGATGGTGAATCCTACGGTCGTTCGTACATTGAGGAATACTTAGGTGACTTACGGTCCCTTGAAAATCTCCAAGAGGCTATCGTCAAGATGTCCATGATTAGCTCTAAGGTTATCGGCTTAGTGAATCCTGCTGGTATCACCCAGCCACGCCGACTGACCAAAGCTCAGACTGGTGACTTCGTTACTGGTCGTCCAGAAGACATCTCGTTCCTCCAACTGGAGAAGCAAGCAGACTTTACTGTAGCTAAAGCCGTAAGTGACGCTATCGAGGCTCGCCTTTCGTTTGCCTTTATGTTGAACTCTGCGGTTCAGCGTACAGGTGAACGTGTGACCGCCGAAGAGATTCGGTATGTAGCTTCTGAACTTGAAGATACTTTAGGTGG‑TGTCTACTCTATCCTTTCTCAAGAATTACAATTGCCTCTGGTACGAGTGCTCTTGAAGCAACTACAAGCCACGCAACAGATTCCTGAGTTACCTAAGGAAGCCGTAGAGCCAACCATTAGTACAGGTCTGGAAGCAATTGGTCGAGGACAAGACCTTGATAA  >  NC_001604.1/20970‑21561`  

                                                                                                                                                                                                                                                                                                       `|`                                                                                                                                                                                                                                                                                                           
`aTCCTAAAGAGGCTTGCCCATACATCCCGATTCGGATGGTCAGACTAGATGGTGAATCCTACGGTCGTTCGTACATT‑AGGAATACTTAGGTGACTTACGGTCCCTTGAAAATCTCCAAGAGGCTATCGTCAAGATGTCCATGATTAGCTCTAAGGTTATCGGCTTAGTGAATCCTGCTGGTATCACCCAGCCACGCCGACTGACCAAAGCTCAGACTGGTGACTTCGTTACTGGTCGTCCAGAAGACATCTCGTTCCTCCAACTGGAGAAGCAAGCAGAATTTACTGTAGCTAACGCCGTa                                                                                                                                                                                                                                                                                                     >  1:281423/1‑301 (MQ=255)`  
 `tCCTAAAGAGGCTTGCCCATACATCCCGATTCGGATGGTCAGACTAGATGGTGAATCCTACGGTCGTTCGTACATTGAGGAATACTTAGGTGACTTACGGTCCCTTGAAAATCTCCAAGAGGCTATCGTCAAGATGTCCATGATTAGCTCTAAGGTTATCGGCTTAGTGAATCCTGCTGGTATCACCCAGCCACGCCGACTGACCAAAGCTCAGACTGGTGACTTCGTTACTGGTCGTCCAGAAGACATCTCGTTCCTCCAACTGGAGAAGCAAGCAGACTTTACTGTAGCTAACGCCGTa                                                                                                                                                                                                                                                                                                     <  1:271271/301‑1 (MQ=255)`  
   `cTAAAGAGGCTTGCCCATACATCCCGATTCGGATGGTCAGACTAGATGGTGAATCCTACGGTCGTTCGTACATTGAGGAATACTTAGGTGACTTACGGTCCCTTGAAAATCTCCAAGAGGCTATCGTCAAGATGTCCATGATTAGCTCTAAGGTTATCGGCTTAGTGAATCCTGCTGGTATCACCCAGCCACGCCGACTGACCAAAGCTCAGACTGGTGACTTCGTTACTGGTCGTCCAGAAGACATCTCGTTCCTCCAACTGGAGAAGCAAGCAGACTTTACTGTAGCTAACGCCGTAAg                                                                                                                                                                                                                                                                                                   <  1:33790/301‑1 (MQ=255)`  
     `aaaGAGGCTTGCCCATACATCCCGATTCGGATGGTCAGACTAGATGGTGAATCCTACGGTCGTTCGTACATTGAGGAATACTTAGGTGACTTACGGTCCCTTGAAAATCTCCAAGAGGCTATCGTCAAGATGTCCATGATTAACTCTAAGGTTATCGGCTTAGTGAATCCTGCTGGTATCACCCAGCCACGCCGACTGACCAAAGCTCAGACTGGTGACTTCGTTACTGGTCGTCCAGAAGACATCTCGTTCCTCCAACTGGAGAAGCAAGCAGACTTTACTGTAGCTAACGCCGTAAGTg                                                                                                                                                                                                                                                                                                 <  1:288863/301‑1 (MQ=255)`  
         `agGCTTGCCCATACATCCCGATTCGGATGGTCAGACTAGATGGTGAATCCTACGGTCGTTCGTACATTGAGGAATACTTAGGTGACTTACGGTCCCTTGAAAATCTCCAAGAGGCTATCGTCAAGATGTCCATGATTAGCTCTAAGGTTATCGGCTTAGTGAATCCTGCTGGTATCACCCAGCCACGCCGACTGACCAAAGCTCAGACTGGTGACTTCGTTACTGGTCGTCCAGAAGACATCTCGTTCCTCCAACTGGAGAAGCAAGCAGACTTTACTGTAGCTAACGCCGTAAGTGACGc                                                                                                                                                                                                                                                                                             <  1:110201/301‑1 (MQ=255)`  
                    `ggcacggtgatgtttataagagacagaGATGGTGAATCCTACGGTCGTTCGTACATTGAGGAAGACTTAGGTGACGTACGGTCGCTTGAAACTCTCCAAGAGGCTATCGTCAGGATGTCCATGATTAGCTCTAAGGTTATCGGCTTAGTGAATCCTGCGGGTATCACCCAGCCACGCCGACTGACCAAAGCTCAGACTGGTGACTTCGTTACTGGTCGTCCAGAAGACATCTCGTTCCTCCAACTGGAGAAGCAAGCAGACTTTACTGTAGCTAACGCCGTAAGTGACGCTATCGCGGATc                                                                                                                                                                                                                                                                                  <  1:250987/275‑1 (MQ=255)`  
                        `tCCCGATACGGATGGTCAGACTAGATGGTGAATCCTACGGTCGTTCGTACATTGAGGAATACTTAGGTGACTTACGGTCCCTTGAAAATCTCCAAGAGGCTATCGTCAAGATGTCCATGATTAGCTCTAAGGTTATCGGCTTAGTGAATCCTGCTGGTATCACCCAGCCACGCCGACTGACCAAAGCTCAGACTGGTGACTTCGTTACTGGTCGTCCAGAAGACATCTCGTTCCTCCAACTGGAGAAGCAAGCAGACTTTACTGTAGCTAACGCCGTAAGTGACGCTATCGCGGCTCGCCt                                                                                                                                                                                                                                                                              >  1:71958/1‑301 (MQ=255)`  
                                              `gacaggTGAATCCTACGGTCGTTCGTACATTGAGGAATACTTAGGTGACTTACGGTCCCTTGAAAATCTCCAAGAGGCTATCGTCAAGAGGTCCATGATTAGCTCTAAGGTTATCGGCTTAGTGAATCCTGCTGGTATCACCCAGCCACGCCGACTGACCAAAGCTCAGACTGGTGACGTCGTTACTGGTCGTCCAGAAGACATCTCGTTCCTCCAACTGGAGAAGCAAGCAGACTTTACTGTAGCTAACGCCGTAAGTGACGCTATCGCGGCTCGCCTTTCGTTTGCCTTTATGTTGAAc                                                                                                                                                                                                                                                        <  1:161720/297‑1 (MQ=255)`  
                                              `aGATGGTGAATCCTACGGTCTTTCGTACATGGAGGAATACTTAGGTGACTTACGGTCCCTTGAAAATCTCCAAGAGGCTATCGTCAAGATGTCCATGATTAGCTCTAAGGTTATCGGCTTAGTGAATCCTGCTGGTATCACCCAGCCACGCCGACTGACCAAAGCTCAGACTGGTGACTTCGTTACTGGTCGTCCAGAAGACATCTCGTTCCTCCAACTGGAGAAGCAAGCAGACTTTACTGTAGCTAACGCCGTAAGTGACGCTATCGCGGCTCGCCTTTCGTTTGCCTTTATGTTGAAc                                                                                                                                                                                                                                                        <  1:30039/301‑1 (MQ=255)`  
                                              `aGATGGGGAATCCTACGGTAGTTAGTAAATTGAGGAATACTTAGGTGACTTACGGTCCCTTGAAAATCTCCAAGAGGCTATCGTCAAGATGTCCATGATTAGCTCTAAGGTTATCGGCTTAGTGAATCCTGCTGGTATCACCCAGCCACGCCGACTGACCAAAGCTCAGACTGGTGACTTCGTTACTGGTCGTCCAGAAGACATCTCGTTCCTCCAACTGGAGAAGCAAGCAGACTTTACTGTAGCAAACGCCGTAAGTGACGCTATCGCGGCTCGCCTTTCGTTTGCCTTTATGTTGAAc                                                                                                                                                                                                                                                        <  1:194916/301‑1 (MQ=255)`  
                                                  `ggTGAATCCTACGGTCGTTCGTACATTGAGGAATACTTAGGTGACTTACGGTCCCTTGAAAATCTCCAAGAGGCTATCGTCAAGATGTCCATGATTAGCTCTAAGGTTATCGGCTTAGTGAATCCTGCTGGTATCACCCAGCCACGCCGACTGACCAAAGCTCAGACTGGTGACTTCGTTACTGGTCGTCCAGAAGACATCTAGTTCCTCCAACTGGAGAAGCAAGCAGACTTTACTGTAGCTAACGCCGTAAGTGACGCTATCGAGGCTCGCCTTTCGTTTGCCTTTATGtctgtctctt                                                                                                                                                                                                                                                    >  1:2162/1‑292 (MQ=255)`  
                                                       `aTCCTACGGTCGTTCGTACATTGAGGAATACTTAGGTGACTTACGGTCCCTTGAAAATCTCCAAGAGGCTATCGTCAAGATGTCCATGATTAGCTCTAAGGTTATCGGCTTAGTGAATCCTGCTGGTATCACCCAGCCACGCCGACTGACCAAAGCTCAGACTGGTGACTTCGTTACTGGTCGTCCAGAAGACATCTCGTTCCTCCAACTGGAGAAGCAAGCAGACTTTACTGTAGCTAACGCCGTAAGTGACGCTATCGCGGCTCGCCTTTCGTTTGCCTTTATGTTGAACTCTGCGGtt                                                                                                                                                                                                                                               >  1:104511/1‑301 (MQ=255)`  
                                                        `tCCTACGGTCGTTCGTACATTGAGGAATACTTAGGTGACTTACGGTCCCTTGAAAATCTCCAAGAGGCTATCGTCAAGATGTCCATGATTAGCTCTAAGGTTATCGGCTTAGTGAATCCTGCTGGTATCACCCAGCCACGCCGACTGACCAAAGCTCAGACTGGTGACTTCGTTACTGGTCGTCCAGAAGACATCTCGTTCCTCCAACTGGAGAAGCAAGCAGACTTTACTGTAGCTAACGCCGTAAGTGACGCTATCGCGGCTCGCCTTTCGTTTGCCTTTATGTTTAACTCTGCGGTTc                                                                                                                                                                                                                                              >  1:257178/1‑301 (MQ=255)`  
                                                        `tCCTACGGTCGTTCGTACAGTGAGGAATACTTAGGTGACTTACGGTCCCTTGAAAATCTCCAAGGGGCTATCGTCAAGATGTCCATGATTAGCTCTAAGGTTATCGGCTTAGTGAATCCTGCTGGTATCGCCCAGCCACGCCGACTGACCAAAGCTCAGACTGGTGACTTCGTTACTGGTCGTCCAGAAGACATCTCGTTCCTCCAACTGGAGAAGCAAGCAGACTTTACTGTAGCTAACGCCGTAAGTGACGCTATCGCGGCTCGCCTTTCGTTTGCCTTTATGTTGAACTCTGCGGTTc                                                                                                                                                                                                                                              <  1:35495/301‑1 (MQ=255)`  
                                                          `cTACGGTCGTTCGTACATTGAGGAATACTTAGGTGACTTACGGTCCCTTGAAAATCTCCAAGAGGCTATCGTCAAGATGTCCATGATTAGCTCTAAGGTTATCGGCTTAGTGAATCCTGCTGGTATCACCCAGCCACGCCGACTGACCAAAGCTCAGACTGGTGACTTCGTTACTGGTCGTCCAGAAGACATCTCGTTCCTCCAACTGGAGAAGCAAGCAGACTTTACTCTAGCTAACGCCGTAAGTGACGCTATCGCTGCTCGCCTTTCGTTTTTCTTTATGTTTAACTCTGCCGTTccc                                                                                                                                                                                                                                            >  1:334433/1‑299 (MQ=255)`  
                                                             `taagagacagtACATTGAGGAATACTTAGGTGACTTACGGTCCCTTGAAAATCTCCAAGAGGCTATCGTCAAGATGTCCATGATTAGCTCTAAGGTTATCGGCTTAGTGAATCCTGCTGGTATCACCCAGCCACGCCGACTGACCAAAGCTCAGACTGGTGACTTCGTTACTGGTCGTCCAGAAGACATCTCGTTCCTCCAACTGGAGAAGCAAGCAGACTTTACTGTAGCTAACGCCGTAAGTGACGCTATCGAGGCTCGCCTTTCGTTTGCCTTTATGTTGAACTCTGCGGTTCAGCGt                                                                                                                                                                                                                                         <  1:15874/292‑1 (MQ=255)`  
                                                               `gTCGTTCGTACATTGAGGGATACTTAGGTGACTTACGGTCCCTTGAAAATCTCCAAGAGGCTATCGTCAAGATGTCCATGATTAGCTCTAAGGTTATCGGCTTAGTGAATCCTGCTGGTATCACCCAGCCACGCCGACTGACCAAAGCTCAGACTGGTGACTTCGTTACTGGTCGTCCAGAAGACATCTCGTTCCTCCAACTGGAGAAGCAAGCAGACTTTACTGTAGCTAACGCCGTAAGTGACGCTATCGCGGCTCGCCTTTCGTTTGCCTTTATGTTGAACTCTGCGGTTCAGCGTAc                                                                                                                                                                                                                                       >  1:14628/1‑301 (MQ=255)`  
                                                               `gTCGTTCGTACATTGAGGAATACTTAGGTGACTTACGGTCCCTTGAAAATCTCCAAGAGGCTATCGTCAAGATGTCCATGATTAGCTCTAAGGTTATCGGCTTAGTGAATCCTGCTGGTATCACCCAGCCACGCCGACTGACCAAAGCTCAGACTGGTGACTTCGTTACTGGTCGTCCAGAAGACATCTCGTTCCTCCAACTGGAGAAGCAAGCAGACTTTACTGTAGCTAACGCCGTAAGTGACGCTATCGCGGCTCGCCTTTCGTTTGCCTTTATGTTGAACTCTGCGGTTCAGCGTAc                                                                                                                                                                                                                                       <  1:119916/301‑1 (MQ=255)`  
                                                                  `tgtgtataagagacagaTACTTAGGTGACTTACGGTCCCTTGAAAATCTCCAAGAGGCTATCGTCAAGATGTCCATGATTAGCTCTAAGGTTATCGGCTTAGTGAATCCTGCTGGTATCACCCAGCCACGCCGACTGACCAAAGCTCAGACTGGTGACTTCGTTACTGGTCGTCCAGAAGACATCTCGTTCCTCCAACTGGAGAAGCAAGCAGACTTTACTGTAGCTAACGCCGTAAGTGACGCTATCGAGGCTCGCCTTTCGTTTGCCTTTATGTTGAACTCTGCGGTTCAGCGTACAgg                                                                                                                                                                                                                                    <  1:235198/285‑1 (MQ=255)`  
                                                                  `gttcgtACATTGAGGAATACTTAGGTGACTTACGGTCCCTTGAAAATCTCCAAGAGGCTATCGTCAAGATGTCCATGATTAGCTCTAAGGTTATCGGCTTAGTGAATCCTGCTGGTATCACCCAGCCACGCCGACTGACCAAAGCTCAGACTGGTGACTTCGTTACTGGTCGTCCAGAAGACATCTCGTTCCTCCAACTGGAGAAGCAAGCAGACTTTACTGTAGCTAACGCCGTAAGTGACGCTATCGCGGCTCGCCTTTCGTTTGCCTTTATGTTGAACTCTGCGGTTCAGCGTACAgg                                                                                                                                                                                                                                    <  1:129694/301‑1 (MQ=255)`  
                                                                   `ttcgtACATTGAGGAATACTTAGGTGACTTACGGTCCCTTGAAAATCTCCAAGAGGCTATCGTCAAGATGTCCATGATTAGCTCTAAGGTTATCGGCTTAGTGAATCCTGCTGGTATCACCCAGCCACGCCGACTGACCAAAGCTCAGACTGGTGACTTCGTTACTGGTCGTCCAGAAGACATCTCGTTCCTCCAACTGGAGAAGCAAGCAGACTTTACTGTAGCTAACGCCGTAAGTGACGCTATCGCGGCTCGCCTTTCGTTTGCCTTTATGTTGAACTCTGCGGTTCAGCGTACAGGt                                                                                                                                                                                                                                   >  1:89477/1‑301 (MQ=255)`  
                                                                   `ttcgtACATTGAGGAATACTTAGGTGACTTACGGTCCCTTGAAAATCTCCAAGAGGCTATCGTCAAGATGTCCATGATTAGCTCTAAGGTTATCGGCTTAGTGAATCCTGCTGGTATCACCCAGCCACGCCGACTGACCAAAGCTCAGACTGGTGACTTCGTTACTGGTCGTCCAGAAGACATCTCGTTCCTCCAACTGGAGAAGCAAGCAGACTTTACTGTAGCTAACGCCGTAAGTGACGCTATCGCGGCTCGCCTTTCGTTTGCCTTTATGTTGAACTCTGCGGTTCAGCGTACAGGt                                                                                                                                                                                                                                   >  1:208066/1‑301 (MQ=255)`  
                                                                    `tcgtACATTGAGGAATACTTAGGTGACTTACGGTCCCTTGAAAATCTCCAAGAGGCTATCGTCAAGATGTCCATGATTAGCTCTAAGGTTATCGGCTTAGTGAATCCTGCTGGTATCACCCAGCCACGCCGACTGACCAAAGCTCAGACTGGTGACTTCGTTACTGGTCGTCCAGAAGACATCTCGTTCCTCCAACTGGAGAAGCAAGCAGACTTTACTGTAGCTAACGCCGTAAGTGACGCTATCGCGGCTCGCCTTTCGTTTGCCTTTATGTTGAACTCTGCGGTTCAGCGTACAGGTg                                                                                                                                                                                                                                  <  1:32072/301‑1 (MQ=255)`  
                                                                    `tcgtACATTGAGGAATACTTAGGTGACTTACGGTCCCTTGAAAATCTCCAAGAGGCTATCGTCAAGATGTCCATGATTAGCTCTAAGGTTATCGGCTTAGTGAATCCTGCTGGTATCACCCAGCCACGCCGACTGACCAAAGCTCAGACTGGTGACTTCGTTACTGGTCGTCCAGAAGACATCTCGTTCCTCCAACTGGAGAAGCAAGCAGACTTTACTGTAGCTAACGCCGTAAGTGACGCTATCGCGGCTCGCCTTTCGTTTGCCTTTATGTTGAACTCTGCGGTTCAGCGTACAGGTg                                                                                                                                                                                                                                  <  1:197694/301‑1 (MQ=255)`  
                                                                          `aTTGAGGAATTCTTAGGTGACTTACGGTCCCTTGAAAATCTCCAAGAGGCTATCGTCAAGATGTCCATGATTAGCTCTAAGGTTATCGGCTTAGTGAATCCTGCTGGTATCACCCAGCCACGCCGACTGACCAAAGCTCAGACTGGTGACTTCGTTACTGGTCGTCCAGAAGACATCTCGTTCCTCCAACTGGAGAAGCAAGCAGACTTTACTGTAGCTAACGCCGTAAGTGACGCTATCGAGGCTCGCCTTTCGTTTGCCTTTATGTTGAACTCTGCGGTTCAGCGTACAGGTGAACgtg                                                                                                                                                                                                                            <  1:295017/301‑1 (MQ=255)`  
                                                                             `gAGGAATACTTAGGTGACTTACGGTCCCTTGAAAATCTCCAAGAGGCTATCGTCAAGATGTCTATGATTAGCTCTAAGGTTATCGGCTTAGTGAATCCTGCTGGTATCACCCAGCCACGCCGACTGACCAAAGCTCAGACTGGTGACTTCGTTACTGGTCGTCCAGAAGACATCTCGTTCCTCCAACTGGAGAAGCAAGCAGACTTTACTGTAGCTAACGCCGTAAGTGACGCTATCGCGGCTCGCCTTTCGTTTGCCTTTATGTTGTACTCTGcctgtatctgttacacatctccgagcc                                                                                                                                                                                                                         >  1:323446/1‑275 (MQ=255)`  
                                                                             `gAGGAATACTTAGGTGACTTACGGTCCCTTGAAAATCTCCAAGAGGCTATCGTCAAGATGTCCATGATTAGCTCTAAGGTTATCGGCTTAGTGAATCCTGCTGGTATCACCCAGCCACGCCGACTGACCAAAGCTCAGACTGGTGACTTCGTTACTGGTCGTCCAGAAGACATCTCGTTCCTCCAACTGGAGAAGCAAGCAGACTTTACTGTAGCTAACGCCGTAAGTGACGCTATCGCGGCTCGCCTTTCGTTTGCCTTTATGTTGAACTCTGCGTTTCCGCGTACAGGTGAACTtgtgg                                                                                                                                                                                                                         >  1:123074/1‑300 (MQ=255)`  
                                                                              `aGGAATACGTAGGTGACTTACGGGCCCTTGAAAATCTCCAAGAGGCTATCGTCAAGATGGCCATGATTAGCTCTAAGGTTATCGGCTTAGTGAATCCTGCTGGTATCACCCAGCCACGCCGACTGACCAAAGCTCAGACTGGTGACTTCGTTACTGGTCGTCCAGAAGACATCTCGTTCCTCCAACTGGAGAAGCAAGCAGACTTTACTGTAGCTAACGCCGTAAGTGACGCTATCGCGGCTCGCCTTTCGTTTGCCTTTATGTTGAACTCTGCGGTTCAGCGTACAGGTGAACGTGGGAc                                                                                                                                                                                                                        <  1:132464/301‑1 (MQ=255)`  
                                                                                       `tAGGTGACTTACGGTCCCTTGAAAATCTCCAAGAGGCTATCGTCAAGATGTCCATGATTAGCTCTAAGGTTATCGGCTTAGTGAATCCTGCTGGTATCACCCAGCCACGCCGACTGACCAAAGCTCAGACTGGTGACTTCGTTACTGGTCGTCCAGAAGACATCTCGTTCCTCCAACTGGAGAAGCAAGCAGACTTTACTGTAGCTAACGCCGTAAGTGACGCTATCGAGGCTCGCCTTTCGTTTGCCTTTATGTTGAACTCTGCGGTTCAGCGTACAGGTGAACctgtctcttatacaca                                                                                                                                                                                                               >  1:274439/1‑289 (MQ=255)`  
                                                                                         `ggTGACTTACGGTCCCTTGAAAATCTCCAAGAGGCTATCGTCATGATGTCCATGATTAGCTCTAAGGTTATCGGCTTAGTGAATCCTGCTGGTATCACCCAGCCACGCCGACTGACCAAAGCTCAGACTGGTGACTTCGTTACTGGTCGTCCAGAAGACATCTCGTTCCTCCAACTGGAGAAGCAAGCAGACTTTACTGTAGCTAACGCCGTAAGTGACGCTATCGCGGCTCGCCTTTCGTTTGCCTTTATGTTGAACTCTGCGGTTCAGCGTACAGGTGAACGTGTGAcctgtctcttat                                                                                                                                                                                                             >  1:309773/1‑291 (MQ=255)`  
                                                                                         `ggTGACTTACGGTCCCTTGAAAATCTCCAAGAGGCTATCGTCAAGATGTCCATGATTAGCTCTAAGGTTATCGGCTTAGTGAATCCTGCTGGTATCACCCAGCCACGCCGACTGACCAAAGCTCAGACTGGTGACTTCGTTACTGGTCGTCCAGAAGACATCTCGTTCCTCCAACTGGAGAAGCAAGCAGACTTTACTGTAGCTAACGCCGTAAGTGACGCTATCGAGGCTCGCCTTTCGTTTGCCTTTATGTTGAACTCTGCGGTTCAGCGTACAGGTGAACGTGTGACCGCCGAAgcga                                                                                                                                                                                                             >  1:312380/1‑301 (MQ=255)`  
                                                                                            `gACTTACGGTCCCTTGAAAATCTCCAAGAGGCTATCGTCAAGATGTCCATGATTAGCTCTAAGGTTATCGGCTTAGTGAATCCTGCTGGTATCACCCAGCCACGCCGACTGACCAAAGCTCAGACTGGTGACTTCGTTACTGGTCGTCCAGAAGACATCTCGTTCCTCCAACTGGAGAAGCAAGCAGACTTTACTGTAGCTAACGCCGTAAGTGACGCTATCGCGGCCCGCCTTTCGTTTGCCTTTATGTTGAACTCTGCGGTTCAGCGTACAGGTGAACGTGTGACCGCCGAAGAGATTc                                                                                                                                                                                                          >  1:202645/1‑301 (MQ=255)`  
                                                                                                `tACGGTCCCTTGAAAAGCTCCAAGAGGCTATCGTCAAGATGTCCATGATTAGCTCTAAGGTTATCGGCTTAGTGAATCCTGCTGGTATCACCCAGCCACGCCGACTGACCAAAGCTCAGACTGGTGACTTCGTTACTGGTCGTCCAGAAGACATCTCGTTCCTCCAACTGGAGAAGCAAGCAGACTTTACTGTAGCTAACGCCGTAAGTGACGCTATCGCGGCTCGCCTTTAGTTTGCCTTTATGTTGAACTCTGCGGTTCAGCGTACAGGTGAACGTGTGACCGCCGAAGAGATTCGGTa                                                                                                                                                                                                      <  1:78722/301‑1 (MQ=255)`  
                                                                                                     `tataagagacagctcCAAGATGCTATCGTCAAGATGTCCATGATTAGCTCTAAGGTTATCGGCTTAGTGAATCCTGCTGGTATCACCCAGCCACGCCGACTGACCAAAGCTCAGACTGGTGACTTCGTTACTGGTCGTCCAGAAGACATCTCGTTCCTCCAACTGGAGAAGCAAGCAGACTTTACTGTAGCTAACGCCGTAAGTGACGCTATCGCGGCTCGCCTTTCGTTTGCCTTTATGTTGAACTCTGCGGTTCAGCGTACAGGTGAACGTGTGACCGCCGAAGAGATTCGGTATGTAg                                                                                                                                                                                                 <  1:59062/289‑1 (MQ=255)`  
                                                                                                     `gcccTTGAAAAACTCCAAGAGGCCATCGTAAAGAAGGCCATGATTAGCTCGAAGGGTATCGGCTTAGGGAAACCTGCTGGTATCACCCAGCCACGCCGACTGACCAAAGCGCAGACTGGGGAATTCGGTACCGGTCGTCCAGAAGACATCTCGTTCCTCCAACTGGAGAAGCAAGCAGACTTTACTGTAGCTAACGCCGTAAGTGACGATATCGCGGCTCGCCTTTCGTTTGCCTTTATGTTGAACTCTGCGGTTCAGCGTGCAGGTGAACGTGTGACCGCCGAAGAGATTCGGTATGTAg                                                                                                                                                                                                 <  1:223360/300‑1 (MQ=255)`  
                                                                                                      `cccTTGAAAATCTCCAAGAGGCTATCGTCAAGATGTCCATGATTAGCTCTAAGGTTATCGGCTTAGTGAATCCTGCTGGTATCACCCAGCCACGCCGACTGACCAAAGCTCAGACTGGTGACTTCGTTACTGGTCGTCCAGAAGACATCTCGTTCCTCCAACTGGAGAAGCAAGCAGACTTTACTGTAGCTAACGCCGTAAGTGACGCTATCGCGGCTCGCCTTTCGTTTGCCTTTATGTTGAACTCTGCGGTTCAGCGTACAGGTGAACGTGTGACCGCCGAAGAGATTCGGTATGTAGc                                                                                                                                                                                                >  1:126651/1‑301 (MQ=255)`  
                                                                                                      `cccTTGAAAATCTCCAAGAGGCTATCGTCAAGATGTCCATGATTAGCTCTAAGGTTATCGGCTTAGTGAATCCTGCTGGTATCACCCAGCCACGCCGACTGACCAAAGCTCAGACTGGTGACTTCGTTACTGGTCGTCCAGAAGACATCTCGTTCCTCCAACTGGAGAAGCAAGCAGACTTTACTGTAGCTAACGCCGTAAGTGACGCTATCGCGGCTCGCCTTTCGTTTGCCTTTATGTTGAACTCTGCGGTTCAGCGTACAGGTGAACGTGTGACCGCCGAAGAGATTCGGTATGTAGc                                                                                                                                                                                                <  1:136520/301‑1 (MQ=255)`  
                                                                                                                 `ctcCAAGAGGCTATCGTCAAGATGTCCATGATTAGCTCTAAGGTTATCGGCTTAGTGAATCCTGCTGGTATCACCCAGCCACGCCGACTGACCAAAGCTCAGACTGGTGACTTCGTTACTGGTCGTCCAGAAGACATCTCGTTCCTCCAACTGGAGAAGCAAGCAGACTTTACTGTAGCTAACGCCGTAAGTGACGCTATCGCGGCTCGCCTTTCGTTTGCCTTTATGTTGAACTCTGCGGTTCAGCGTACAGGTGAACGTGTGACCGCCGAAGAGATTCGGTATGTAGCTTCTGAACCTg                                                                                                                                                                                     >  1:4355/1‑301 (MQ=255)`  
                                                                                                                 `atcCAAGAGGCTATCGTCAAGATGACCATGATTAGCTCTAAGGTTATCGGCTTAGTGAATCCTGCTGGTATCACCCCGCCACGCCGACTTACCAACGCTCAGACTAGTGAATTCGTTCCTGCTCGTCCAGAAGACATCTCGTTCCTCCAACAGGAAAAGCAAGCAGACTTTACTCTAGCTAACGCCGTAAGTGACGCTATCCCGGCTCGCCTTTCGTTTGCCTTTATGTTAGACTCTGCGGTTCACCTTACAGGTGTACGTGTGACCGCCGTAGCGATTCGGTATCTAGccccttacattt                                                                                                                                                                                     >  1:168850/2‑290 (MQ=255)`  
                                                                                                                             `aTCGTCAAGATGTCCATGATTAGCTCTAAGGTTATCGGCTTAGTGAATCCTGCTGGTATCACCCAGCCACGCCGACTGACCAAAGCTCAGACTGGTGACTTCGTTACTGGTCGTCCAGAAGACATCTCGTTCCTCCAACTGGAGAAGCAAGCAGACTTTACTGTAGCTAACGCCGTAAGTGACGCTATCGCGGCTCGCCTTTCGTTTGCCTTTATGTTGAACTCTGCGGTTCAGCGTACAGGTGAACGTGTGACCGCCGAAGAGATTCGGTATGTAGCTTCTGAACTTGAAGATACTTTAg                                                                                                                                                                         >  1:38600/1‑301 (MQ=255)`  
                                                                                                                              `tCGTCAAGATGTCCATGATTAGCTCTAAGGTTATCGGCTTAGTGAATCCTGCTGGTATCACCCAGCCACGCCGACTGACCAAAGCTCAGACTGGTGACTTCGTTACTGGTCGTCCAGAAGACATCTCGTTCCTCCTACTGGAGAAGCAAGCAGACTTTACTGTAGCTAACGCCGTAAGTGACGCTATCGAGGCTCGCCTTTCGTTTGCCTTTATGTTGAACTCTGCGGTTCAGCGTCCAGGTGAACGTGTGACCGCCGAAGAGATTCGGTATGTAGCTTCTGAACTTGAAGATACTTTAgg                                                                                                                                                                        >  1:219198/1‑301 (MQ=255)`  
                                                                                                                                 `cagaGATGTCCATGATTAGCTCTAAGGTTATCGGCTTAGTGAATCCTGCTGGTATCACCCAGCCACGCCGACTGACCAAAGCTCAGACTGGTGACTTCGTTACTGGTCGTCCAGAAGACATCTCGTTCCTCCAACTGGAGAAGCAAGCAGACTTTACTGTAGCCAACGCCGTAAGTGACGCTATCGAGGCTCGCCTTTCGTTTGCCTTTATGTTGAACTCTGCGGTTCAGCGTACAGGTGAACGTGTGACCGCCGAAGAGATTCGGTATGTAGCTTCTGAACTTGAAGATACTTTAggtgg                                                                                                                                                                     <  1:140146/298‑1 (MQ=255)`  
                                                                                                                                     `caggTCCATGATTAGCTCTAAGGTTATCGGCTTAGTGAATCCTGCTGGTATCACCCAGCCACGCCGACTGACCAAAGCTCAGACTGGTGACTTCGTTACTGGTCGTCCAGAAGACATCTCGTTCCTCCAACTGGAGAAGCAAGCAGACTTTACTGTAGCTAACGCCGTAAGTGACGCTATCGCGGCTCGCCTTTCGTTTGCCTTTATGTTGAACTCTGCGGTTCAGCGTACAGGTGAACGTGTGACCGCCGAAGAGATTCGGTATGTAGCTTCTGAACTTGAAGATACTTTAGGTGG‑TGTc                                                                                                                                                                <  1:165460/298‑1 (MQ=255)`  
                                                                                                                                        `gTCCATGATTAGCTCTAAGGTTATCGGCTTAGTGAATCCTGCTGGTATCACCCAGCCACGCCGACTGACCAAAGCTCAGACTGGTGACTTCGTTACTGGTCGTCCAGAAGACATCTCGTTCCTCCAACTGGAGAAGCAAGCAGACTTTACTGTAGCTAACGCCGTAAGTGACGCTATCGCGGCTCGCCTTTCGTTTGCCTTTATGTTGAACTCTGCGGTTCAGCGTACAGGTGAACGTGTGACCGCCGGAGAGAGTCGGTATGTAGCTTCTGAACTTGAAGATACTTTAGGTGG‑TGTCtct                                                                                                                                                             >  1:18552/1‑299 (MQ=255)`  
                                                                                                                                        `gTCCATGATTAGCTCTAAGGTTATCGGCTTAGTGAATCCTGCTGGTATCACCCAGCCACGCCGACTGACCAAAGCTCAGACTGGTGACTTCGTTACTGGTCGTCCAGAAGACATCTCGTTCCTCCAACTGGAGAAGCAAGCAGACTTTACTGTAGCTAACGCCGTAAGTGACGCTATCGCGGCTCGCCTTTCGTTTGCCTTTATGTTGAACTCTGCGGTTCAGCGTACAGGTGAACGTGTGACCGCCGAAGAGATTCGGTATGTAGCTTCTGAACTTGAAGATACTTTAggtctgtctctt                                                                                                                                                              >  1:212264/1‑292 (MQ=255)`  
                                                                                                                                        `gTCCATGATTAGCTCTAAGGTTATCGGCTTAGTGAATCCTGCTGGTATCACCCAGCCACGCCGACTGACCAAAGCTCAGACTGGTGACTTCGTTACTGGTCGTCCAGAAGACATCTCGTTCCTCCAACTGGAGAAGCAAGCAGACTTTACTGTAGCTAACGCCGTAAGTGACGCTATCGCGGCTCGCCTTTCGTTTGCCTTTATGTTGAACTCTGCGGTTCAGCGTACAGGTGAACGTGTGACCGCCGAAGAGATTCGGTATGTAGCTTCTGAACTTGAAGATACTTTAGGTGG‑TGTCTAc                                                                                                                                                             >  1:84606/1‑301 (MQ=255)`  
                                                                                                                                              `gATTAGCTCTAAGGTTATCGGCTTAGTGAATCCTGCTGGTATCACCCAGCCACGCCGACTGACCAAAGCTCAGACTGGTGACTTCGTTACTGGTCGTCCAGAAGACATCTCGTTCCTCCAACTGGAGAAGCAAGCAGACTTTACTGTAGCTAACGCCGTTAGTGACGCTATCGCGGCTCGCCTTTCGTTTGCCTTTATGTTGAACTCTGCGGTTCAGCGTACAGGTGAACGTGTGACCGCCGAAGAGATTCGGTATGTAGCTTCTGAACTTGAAGATACTTTAGGTGGCTGTCtcttatac                                                                                                                                                        >  1:267439/1‑294 (MQ=255)`  
                                                                                                                                               `acaggccctAAGGTTATCGGCTTAGTGAATCCTGCTGGTATCACCCAGCCACGCCGACTGACCAAAGCTCAGACTGGTGACTTCGTTACTGGTCGTCCAGAAGACATCTCGTTCCTCCAACTGGAGAAGCAAGCAGACTTTACTGTAGCTAACGCCGTAAGTGACGCTATCGCGGCTCGCCTTTCGTTTGCCTTTATGTTGAACTCTGCGGTTCAGCGTACAGGTGAACGTGTGACCGCCGAAGAGATTCGGTATGTAGCTTCTGAACTTGAAGATACTTTAGGTGG‑TGTCTACTCTATcc                                                                                                                                                      <  1:277873/294‑1 (MQ=255)`  
                                                                                                                                                   `gCTCTAAGGTTATCGGCTTAGTGAATCCTGCTGGTATCACCCAGCCACGCCGACTGACCAAAGCTCAGACTGGTGACTTCGTTACTGGTCGTCCAGAAGACATCTCGTTCCTCCAACTGGAGAAGCAAGCAGACTTTACTGTAGCTAACGCCGTAAGTGACGCTATCGCGGCTCGCCTTTCGTTTGCCTTTATGTTGCACTCTGCGGTTCAGCGTACAGGTGAACGTGTGACCGCCGAAGAGCTTCGGTATGTAGCTTCTGAACTTGAAGATACTTTAGGTGG‑TTTCTACTCTATCCtttt                                                                                                                                                  >  1:229819/1‑300 (MQ=255)`  
                                                                                                                                                   `gCTCTAAGGTTATCGGCTTAGTGAATCCTGCTGGTATCACCCAGCCACGCCGACTGACCAAAGCTCAGACTGGTGACTTCGTTACTGGTCGTCCAGAAGACATCTCGTTCCTCCAACTGGAGAAGCAAGCAGACTTTACTGTAGCTAACGCCGTAAGTGACGCTATCGCGGCTCGCCTTTCGTTTGCCTTTATGTTGAACTCTGCGGTTCAGCGTACAGGTGAACGTGTGACCGCCGAAGAGATTCGGTATGTAGCTTCTGAACTTGAAGATACTTTAGGTGG‑TGTCTACTCTATCCTTtc                                                                                                                                                  >  1:205383/1‑301 (MQ=255)`  
                                                                                                                                                          `ggTTATCGGCTTAGTGAATCCTGCTGGTATCACCCAGCCACGCCGACTGACCAAAGCTCAGACTGGTGACTTCGTTACTGGTCGTCCAGAAGACATCTCGTTCCTCCAACTGGAGAAGCAAGCAGACTTTACTGTAGCTAACGCCGTAAGTGACGCTATCGCGGCTCGCCTTTCGTTTGCCTTTATGTTGAACTCTGCGGTTCAGCGTACAGGTGAACGTGTGACCGCCGAAGAGATTCGGTATGTAGCTTCTGACCGTGAAGATACTTTAGGTGG‑TTTCTACTCTATCCTTTCTCAATaa                                                                                                                                           >  1:224987/1‑301 (MQ=255)`  
                                                                                                                                                             `tATCGGCTTAGTGAATCCTGCTGGTATCACCCAGCCACGCCGACTGACCAAAGCTCAGACTGGTGACTTCGTTACTGGTCGTCCAGAAGACATCTCGTTCCTCCAACTGGAGAAGCAAGCAGACTTTACTGTAGCTAACGCCGTAAGTGACGCTATCGCGGCTCGCCTTTCGTTTGCCTTTATGTTGAACTCTGCGGTTCAGCGTACAGGTGAACGTGTGACCGCCGAAGAGATTCGGTATGTAGCTTCTGAACTTGAAGATACTTTAGGTGG‑TGTCTACTCTATCCTTTCTCAAGAATTa                                                                                                                                        >  1:68645/1‑301 (MQ=255)`  
                                                                                                                                                                `tataagagacagtCCTGCTGGTATCACCCAGCCACGCCGACTGACCAAAGCTCAGACTGGTGACTTCGTTACTGGTCGTCCAGAAGACATCTCGTTCCTCCAACTGGAGAAGCAAGCAGACTTTACTGTAGCTAACGCCGTAAGTGACGCTATCGCGGCTCGCCTTTCGTTTGCCTTTATGTTGAACTCTGCGGTTCAGCGTACAGGTGAACGTGTGACCGCCGAAGAGATTCGGTATGTAGCTTCTGAACTTGAAGATACTTTAGGTGG‑TGTCTACTCTATCCTTTCTCAAGAATTACaa                                                                                                                                     <  1:81229/289‑1 (MQ=255)`  
                                                                                                                                                                `gggCTTAGTGAAGCCTGCTGGGATCACCCAGCCACGCCGACTGACCAAAGCTCAGACTGGTGACTTCGTTACTGGTCGTCCAGAAGACATCTCGTTCCTCCAACTGGAGAAGCAAGCAGACTTTACTGTAGCTAACGCCGTAAGTGACGCTAGCGCGGCTCGCCTTTCGTTTGCCTTTATGTTGAACGCTGCGGTTCAGCGTACAGGTGAACGTGTGACCGCCGAAGAGATGCGGTATGTAGCGGCGGAACTTGAAGATACTTTAGGTGG‑TGTCTACTCTATCCTTTCTCAAGAATTACaa                                                                                                                                     <  1:180851/300‑1 (MQ=255)`  
                                                                                                                                                                `cGGCTTAGTGAATCCTGCTGGTATCCCCCAGCCACGCCGACTGACCAAAGCTCAGACTTGTGACTTCGTTACGGGTCGTCCAGAAGACATCTCGTTCCTCCAACTGGAGAAGCAAGCAGACTTTACTGTAGCTAACGCCGTAAGTGACGCTATCGCGGCTCGCCTTTCGTTTGCCTTTATGTTGAACTCTGCGGTTCAGCGTACAGGTGAACGTGTGACCGCCGAAGAGATTCGGTATGTAGCTTCTGAACTTGAAGATACTTTAGGTGG‑TGTCTACTCTATCCTTTCTCAAGAATTACaa                                                                                                                                     <  1:92469/301‑1 (MQ=255)`  
                                                                                                                                                                 `gtataagagacagctgctgGTATCACCCAGCCACGCCGACTGACCAAAGCTCAGACTGGTGACTTCGTTACTGGTCGTCCAGAAGACATCTCGTTCCTCCAACTGGAGAAGCAAGCAGACTTTACTGTAGCTAACGCCGTAAGTGACGCTATCGCGGCTCGCCTTTCGTTTGCCTTTATGTTGAACTCTGCGGTTCAGCGTACAGGTGAACGTGTGACCGCCGAAGAGATTCGGTATGCAGCTTCTGAACTTGAAGATACTTTAGGTGG‑TGTCTACTCTATCCTTTCTCAAGAATTAGAAt                                                                                                                                    <  1:178684/288‑1 (MQ=255)`  
                                                                                                                                                                 `ggCTTAGTGAATCCTGCTGGTATCACCCAGCCACGCCGACTGACCAAAGCTCAGACTGGTGACTTCGTTACTGGTTGTCCAGAAGACATCTCGTTCCTCCAACTGGAGAAGCAAGCAGACTTTACTGTAGCTAACGCCGTAAGTGACGCTATCGCGGCTCGCCTTTCGTTTGCCTTTATGTTGAACTCTGCGGTTCAGCGTACAGGTGAACGTGTGACCGCCGAAGAGATTCGGTATGTAGCTTCTGAACTTGAAGATACTTTAGGTGG‑TGTCTACTCTATCCTTTCTCAAGAATTACAAt                                                                                                                                    >  1:307764/1‑301 (MQ=255)`  
                                                                                                                                                                 `ggCTTAGTGAATCCTGCTGGTATCACCCAGCCACGCCGACTGACCAAAGCTCAGACTGGTGACTTCGTTACTGGTCGTCCAGAAGACATCTCGTTCCTCCAACTGGAGAAGCAAGCAGACTTTACTGTAGCTAACGCCGTAAGTGACGCTATCGCGGCTCGCCTTTCGTTTGCCTTTATGTTGAACTCTGCGGTTCAGCGTACAGGTGAACGTGTGACCGCCGAAGAGATTCGGTATGTAGCTTCTGAACTTGAAGATACTTTAGGTGG‑TGTCTActcctgtctcttatacacatctccga                                                                                                                                    >  1:253247/1‑278 (MQ=255)`  
                                                                                                                                                                 `ggCTTAGTGAATCCTGCTGGTATCACCCAGCCACGCCGACTGACCAAAGCTCAGAATGGTGACTTCGTTACTGGTCGTCCAGAAGACATCTCGTTCCTCCAACTGGAGAAGCAAGCAGACTTTACTGTAGCTAACGCCGTAAGTGACGCTATCGCGGCTCGCCTTTCGTTTGCCTTTATGTTGAACTCTGCGGTTCAGCGTACAGGTGAACGTGTGACCGCCGAAGAGATTCGGTATGTAGCTTCTGAACTTGAAGATACTTTAGGTGG‑TGTCTACTCTATCCTTTCTCAAGAATTACAAt                                                                                                                                    <  1:155197/301‑1 (MQ=255)`  
                                                                                                                                                                       `gTGAATCCTGCTGGTATCACCCAGCCACGCCGACTGACCAAAGCTCAGACTGGTGACTTCGTTACTGGTCGTCCAGAAGACATCTCGTTCCTCCAACTGGAGAAGCAAGCAGACTTTACTGTAGCTAACGCCGTAAGTGACGCTATCGCGGCTCGCCTTTCGTTTGCCTTTATGTTGAACTCTGCGGTTCAGCGTACAGGTGAACGTGTGACCGCCGAAGAGATTCGGTATGTAGCTTCTGAACTTGAAGATACTTTAGGTGG‑TGTCTACTCTATCCTTTCTCAAGAATTACAATTGCctc                                                                                                                              <  1:292814/301‑1 (MQ=255)`  
                                                                                                                                                                           `aTCCTGCTGGTATCACCCAGCCACGCCGACTGACCAAAGCTCAGACTGGTGACTTCGTTACTGGTCGTCCAGAAGACATCTCGTTCCTCCAACTGGAGAAGCAAGCAGACTTTACTGTAGCTAACGCCGTAAGTGACGCTATCGCGGCTCGCCTCTCGTTTGCCTTTATGTTGAACTCTGCGGTTCAGCGTACAGGTGAACGTGTGACCGCCGAAGAGATTCGGTATGTAGCTTCTGAACTTGAAGATACTTTAGGTGG‑TGTCTACTCTATCCTTTCTCAAGAATTAAAATTGCCTCTGGt                                                                                                                          >  1:345590/1‑301 (MQ=255)`  
                                                                                                                                                                           `aTCCTGCTGGTATCACCCAGCCACGCCGACTGACCAAAGCTCAGACTGGTGACTTCGTTACTGGTCGTCCAGAAGACATCTCGTTCCTCCAACTGGAGAAGCAAGCAGACTTTACTGTAGCTAACGCCGTAAGTGACACTATCGCGGCTCGCCTTTCGTTTGCCTTTATGTTGAACTCTGCGGTTCAGCGTACAGGTGAACGTGTGACCGCCGAAGAGATTCGGTATGTAGCTTCTGAACTTGAAGATACTTTAGGTGG‑TGTCTACTCTATCCTTTCTCAAGAATTACAATTGCCTCTGGt                                                                                                                          >  1:142947/1‑301 (MQ=255)`  
                                                                                                                                                                            `tCCTGCTGGTATCACCCAGCCACGCCGACTGACCAAAGCTCAGACTGGTGACTTCGTTACTGGTCGTCCAGAAGACATCTCGTTCCTCCAACTGGAGAAGCAAGCAGACTTTACTGTAGCTAACGCCGTAAGTGACGCTATCGAGGCTCGCCTTTCGTTTGCCTTTATGTTGAACTCTGCGGTTCAGCGTACAGGTGAACGTGTGACCGCCGAAGAGATTCGGTATGTAGCTTCTGAACTTGAAGATACTTTAGGTGG‑TGTCTACTCTATCCTTTCTCAAGAATTACAATTGCCTCTGGTa                                                                                                                         <  1:50623/301‑1 (MQ=255)`  
                                                                                                                                                                              `ctgctgGTATCACCCAGCCACGCCGACTGACCAAAGCTCAGACTGGTGACTTCGTTACTGGTCGTCCAGAAGACATCTCGTTCCTCCAACTGGAGAAGCAAGCAGACTTTACTGTAGCTAACGCCGTAAGTGACGCTATCGCGGCTCGCCTTTCGTTTGCCTTTATGTTGAACTCTGCGGTTCAGCGTACAGGTGAACGTGTGTCCTCCGAAGAGATTCGGTATGTAGCTTCTGAACTTGAAGATACTTTAGGTGG‑TGTCTACTCTATCCTTTCTCAAGAATTACAATTTCCTCTGGTACg                                                                                                                       >  1:318045/1‑301 (MQ=255)`  
                                                                                                                                                                                            `cAGCCACGCCGACTGACCAAAGCTCAGACTGGTGACTTCGTTACTGGTCGTCCAGAAGACATCTCGTTCCTCCAACTGGAGAAGCAAGCAGACTTTACTGTAGCTAACGCCGTAAGTGACGCTATCGAGGCTCGCCTTTCGTTTGCCTTTATGTTGAACTCTGCGGTTCAGCGTACAGGTGAACGTGTGACCGCCGAAGAGATTCGGTATGTAGCTTCTGAACTTGAAGATACTTTAGGTGG‑TGTCTACTCTATCCTTTCTCAAGAATTACAATTGCCTCTGGTACGAGTGCTCTTGAAga                                                                                                         >  1:285004/1‑300 (MQ=255)`  
                                                                                                                                                                                                 `aCGCCGACTGACCAAAGGTCAGACTGGTGACTTCGTTACTGGTCGTCCAGAAGTCATCTCGTTCCTCCAACTGGAGAAGCAAGCAGACTTTACTGTAGCTAACGCCGTAAGTGACGCTATCGAGGCTCGCCTTTCGTTTGCCTTTATGTTGAACTCTGCGGTTCAGCGTACAGGTGAACGTGTGACCGCCGAAGAGATTCGGTATGTAGCTTCTGAACTTGAAGATACTTTAGGTGG‑TGTCTACTCTATCCTTTCTCAAGAATTACAATTGCCTCTGGTACGAGTGCTCTTGAAGCAACTa                                                                                                    <  1:183300/301‑1 (MQ=255)`  
                                                                                                                                                                                                  `cGCCGACTGACCAAAGCTCAGACTGGTGACTTCGTTACTGGTCGTCCAGAAGACATCTCGTTCCTCCAACTGGAGAAGCAAGCAGACTTTACTGTAGCTAACGCCGTAAGTGACGCTATCGCGTCTCGCCTTTCGTTTGCCTTTATGTTGAACTCTGCGGTTCAGTGTACAGGTGAACGTGTGACCGCCGATAAGATTCGGTATGGAGCATTTGAACTTCAAGATACTTTAGGTGG‑TGTCTACTCTCTCCTTTTTCAAGAATTACAATTGCCTCTAGTACGAGGGCTCTTGAAGCAACTAc                                                                                                   >  1:233299/1‑301 (MQ=255)`  
                                                                                                                                                                                                  `cGCCGACTGACCAAAGCTCAGACTGGTGACTTCGTTACTGGTCGTCCAGAAGACATCTCGTTCCTCCAACTGGAGAAGCAAGCAGACTTTACTGTAGCTAACGCCGTAAGTGACGCTATCGCGGCTCGCCTTTCGTTTGCCTTTATGTTGAACTCTGCGGTTCAGCGTACAGGTGAACGTGTGACCGCCGAAGAGATTCGGTATGTAGCTTCTGAACTTGAAGATACTTTAGGTGG‑TGTCTACTCTATCCTTTCTCAAGAATTACAATTGCCTCTGGTACGAGTGCTCTTGAAGCAACTAc                                                                                                   >  1:200490/1‑301 (MQ=255)`  
                                                                                                                                                                                                            `ccAAAGCTCAGACTGGTGACTTCGTTACTGGTCGTCCAGAAGACATCTCGTTCCTCCAACTGGAGAAGCAAGCAGACTTTACTGTAGCTAACGCCGTAAGTGACGCTATCGCGGCTCGCCTTTCGTTTGCCTTTATGTTGAACTCTGCGGTTCAGCGTACAGGTGAACGTGTGACCGCCGAAGAGATTCGGTATGTAGCTTCTGAACTTGAAGATACTTTAGGTGG‑TGTCTACTCTATCCTTTCTCAAGAATTACAATTGCCTCTGGTACGAGTGCTCTTGAAGCAACTACAAGccctgtc                                                                                         >  1:117263/1‑296 (MQ=255)`  
                                                                                                                                                                                                                `aGCTCAGACTGGTGACTTCGTTACTGGTCGTCCAGAAGACATCTCGTTCCTCCAACTGGAGAAGCAAGCAGACTTTACTGTAGCTAACGCCGTAAGTGACGCTATCGCTGCTCGCCTTTCGTTTGCCTTTATGTTGAACTCTGCGGTTCAGCGTACAGGTGAACGTGTGACCGCCGAAGAGATTCGGTATGTAGCTTCTGAACTTGAAGATACTTTCGGTGG‑TGTCTACTCTATCCTTTCTCAAGAATTACAATTGCCTCTGGTACGAGTGCTCTTGAAGCAACTACAAGccctgtctctt                                                                                     >  1:176866/1‑292 (MQ=255)`  
                                                                                                                                                                                                                `aGCTCAGACTGGTGACTTCGTTACTGGTCGTCCAGAAGACATCTCGTTCCTCCAACTGGAGAAGCAAGCAGACTTTACTGTAGCTAACGCCGTAAGTGACGCTATCGCGGCTCGCCTTTCGTTTGCCTTTATGTTGAACTCTGCGGTTCAGCGTACAGGTGAACGTGTGACCGCCGAAGAGATTCGGTATGTAGCTTCTGAACTTGAAGATACTTTAGGTGG‑TGTCTACTCTATCCTTTCTCAAGAATTACAATTGCCTCTGGTACGAGTGCTCTTGAAGCAACTACAAGCCACGCAACAg                                                                                     >  1:304321/1‑301 (MQ=255)`  
                                                                                                                                                                                                                    `cAGACTGGTGACTTCGTTACTGGTCGTCCAGAAGACATCTCGTTCCTCCAACTGGAGAAGCAAGCAGACTTTACTGTAGCTAACGCCGTAAGTGACGCTATCGCGGCTCGCCTTTCGTTTGCCTTTATGTTGAACTCTGTGGTTCAGCGTACAGGTGAACGTGTGACCGCCGAAGAGATTCGGTATGTAGCTTCTGAACTTGAAGATACTTTAGGTGG‑TGTCTACTCTATCCTTTCTCAAGAATTACAATTGCCTCTGGTACGAGTGCTCTTGAAGCAACTACAAGCCACGCAACAGATTc                                                                                 <  1:10021/301‑1 (MQ=255)`  
                                                                                                                                                                                                                          `ggTGTCTTCGTTACTGGTCGTCCAGAAGACATCTCGTTCCTCCAGCTGGAGAAGCAAGCAGACTTTACTTTAGCTAACGCCGTAAGTGACGCTATCGCGGCTCGCCTTTCGTTTGCCTTTATGTTGAACTCTGCGGTTCAGCGTACAGGTGAACGTGTGACCGCCGAAGAGATTCGGTATGTAGCTTCTGAACTTGAAGATACTTTAGGTGG‑TGTCTACTCTATCCTTTCTCAAGAATTACAATTGCCTCTGGTACGAGTGCTCTTGAAGCAACTACAAGCCACGCAACAGATTCCTGAGt                                                                           <  1:56177/301‑1 (MQ=255)`  
                                                                                                                                                                                                                          `ggTGACTTCGTTACTGGTCGTCCAGAAGACATCTCGTTCCTCCAACTGGAGAAGCAAGCAGACTTTACTGTAGCTAACACCGTAAGTGACGCTATCGCGGCTCGCCTTTCGTTTGCCTTTATGTTGAACTCTGCGGTTCAGCGTACAGGTGAACGTGTGACCGCCGAAGAGATTCGGTATGTAGCTTCTGAACTTGAAGATACTTTAGGTGG‑TGTCTACTCTATCCTTTCTCAAGAATTACAATTGCCTCTGGTACGAGTGCTCTTGAAGCAACTACAAGCCACGCAACAGATTCCTGAGt                                                                           >  1:107620/1‑301 (MQ=255)`  
                                                                                                                                                                                                                               `cTTCGTTACTGGTCGTCTAGAAGACATCTCGTTCCTCCAACTGGAGAAGCAAGCAGACTTTACTGTAGCTAACGCCGTAAGTGACGCTATCGCGGCTCGCCTTTCGTTTGCCTTTATGTTGAACTCTGCGGTTCAGCGTACAGGTGAACGTGTGACCGCCGAAGAGATTCGGTATGTAGCTTCTGAACTTGAAGATACTTTAGGTGG‑TGTCTACTCTATCCTTTCTCAAGAATTACAATTGCCTCTGGTACGAGTGCTCTTGAAGCAACTACAAGCCACGCAACAGATTCCTGAGTTACCt                                                                      >  1:62028/1‑301 (MQ=255)`  
                                                                                                                                                                                                                               `cTTCGTTACTGGTCGTCCAGAAGACATCTCGTTCCTCCAACTGGAGAAGCAAGCAGACTTTACTGTAGCTAACGCCGTAAGTGACGCTATCGCGGCTCGCC‑TTCGTTTGCCTTTATGTTGAACTCTGCGGTTCAGCGTACAGGTGAACGTGTGACCGCCGAAGAGATTCGGTATGTAGCTTCTGAACTTGAAGATACTTTAGGTGG‑TGTCTACTCTATCCTTTCTCAAGAATTACAATTGCCTCTGGTACGAGTGCTCTTGAAGCAACTACAAGCCACGCAACAGATTCTTGAGTTACCTa                                                                     >  1:238936/1‑301 (MQ=255)`  
                                                                                                                                                                                                                                   `gTTACTGGTCGTCCAGAAGACATCTCGTTCCTCCAACTGAAGAAGCAAGCAGACTTTACTGTAGCTAACGCCGTAAGTGACGCTATCGAGGCTCACCTTTCGTTTGCCTTTATGTTGAACTCTGCGGTTCAGCGTACAGGTGAACGTGTGACCGCCGAAGAGATTCGGTATGTAGCTTCTGAACTTGAAGATACTTTAGGTGG‑TGTCTACTCTATCCTTTCTCAAGAATTACAATTGCCTCTGGTACGAGTGCTCTTGAAGCAACTACAAGCCACGCAACAGATTCCTGAGTTACCTAAgg                                                                  >  1:326658/1‑301 (MQ=255)`  
                                                                                                                                                                                                                                    `ttACTGGTCGTCCAGAAGACATCTCGTTCCTCCAACTGGAGAAGCAAGCAGACTTTACTGTAGCTAACGCCGTAAGTGACGCTATCGCGGCTCGCCTTTCGTTTGCCTTTATGTTGAACTCTGCGGTTCAGCGTACAGGTGAACGTGTGACCGCCGAAGAGATTCGGTATGTAGCTTCTGAACTTGAAGATACTTTAGGTGG‑TGTCTACTCTATCCTTTCTCAAGAATTACAATTGCCTCTGGTACGAGTGCTCTTGAAGCAACTACAAGCCACGCAACAGATTCCTGAGTTACCTAAGGa                                                                 <  1:264541/301‑1 (MQ=255)`  
                                                                                                                                                                                                                                       `cTGGTCGTCCAGAAGACATCTCGGTCCTCCAACTGGAGAAGCAAGCAGACTTTACTGTAGCTAACGCCGTAAGTGACGCTATCGCGGCTCGCCTTTCGTTTGCCTTTATGTTGAACTCTGCGGTTCAGCGTACAGGTGAACGTGTGACCGCCGAAGAGATTCGGTATGTAGCTTCTGAACTTGAAGATACTGTAGGTGG‑TGTCTACTCTATCCTTTCTCAAGAATTACAAGTGCCTCTGGTACAAGTGCTCTTGAAGCAACTACAAGCCACGCAACAGATTCCTGAGTTACCTAAGGAAGc                                                              <  1:47262/301‑1 (MQ=255)`  
                                                                                                                                                                                                                                        `tGGTCGTCCCGAAGACATCTCGCTCCTCCAACTGGAGAAGCAAGCAGACTTTACTGTAGCTAACGGCGTAAGTGACGCTATCGCGGCTCGCCTTTCGTTTGCCTTTATGTTGAACTCGGCGGTTCAGCGTACAGGTGAACGTGTGACCGCCGAAGAGATTCGGTATGTAGCTTCTGAACTTGAAGATACTTTAGGTGG‑TGTCTACTCTATCCTTTCTCAAGAATTACAATTGCCTCTGGTACGAGTGCTCTTGAAGCAACTACAAGCCACGCAACAGATTCCTGAGTTACCTAAGGAAGcc                                                             <  1:41593/301‑1 (MQ=255)`  
                                                                                                                                                                                                                                        `tGGTCGTCCAGAAGACATCTCGTTCCTCCAACTGGAGAAGCAAGCAGACGTTACTGTAGCTAACGCCGTAAGTGACGCTATCGCGGCTCGCCTTTCGTTTGCCTTTATGTTGAACTCTGCGGTTCAGCGTACAGGTGAACGTGTGACCGCCGAAGAGATTCGGTATGTAGCTTCTGAACTTGAAGATACTTTAGGTGG‑TGTCTACTCTATCCTTTCTCAAGAATTACAATTGCCTCTGGTACGAGTGCTCTTGAAGCAACTACAAGCCACGCAACAGATTCCTGAGTTACCTAAGGAAGcc                                                             <  1:1894/301‑1 (MQ=255)`  
                                                                                                                                                                                                                                            `cgtcCAGAAGACATCTCGTTCCTCCAACTGGAGAAGCAAGCAGACTTTACTGTAGCTAACGCCGTAAGTGACGCTATCGCGGCTCGCCTTTCGTTTGCCTTTATGTTGAACTCTGCGGTTCAGCGTACAGGTGAACGTGTGACCGCCGAAGAGATTCGGTATGTAGCTTCTGAACTTGAAGATACTTTAGGTGG‑TGTCTACTCTATCCTTTCTCAAGAATTACAATTGCCTCTGGTACGAGTGCTCTTGAAGCAACTACAAGCCACGCAACAGATTCCTGAGTTACCTAAGGAAGCCGTag                                                         <  1:192203/301‑1 (MQ=255)`  
                                                                                                                                                                                                                                             `gtcCAGAAGACATCTCGTTCCTCCAACTGGAGAAGCAAGCAGACTTTACTGTAGCTAACGCCGTAAGTGACGCTATCGCGGCTCGCCTTTCGTTTGCCTTTATGTTGAACTCTGCGGTTCAGCGTACAGGTGAACGTGTGACCGCCGAAGAGATTCGGTATGTAGCTTCTGAACTTGAAGATACTTTAGGTGG‑TGTCTACTCTATCCTTTCTCAAGAATTACAATTGCCTCTGGTACGAGTGCTCTTGAAGCAACTACAAGCCACGCAACAGATTCCTGAGTTACCTAAGGAAGCCGTaga                                                        >  1:75482/1‑301 (MQ=255)`  
                                                                                                                                                                                                                                              `tcCAGAAGCCATCTCGTTTCTCCAACTGGATAAGCAAGCAGACTTTACTGTAGCTAACGCCGTAAGTGACGCTATCGCGGCTCGCCTTTCGTTTGCCTTTATGTTGTACGCTGCGGTTCAGCGTACAAGTGAACGTGTGACCGCCGAAGAGATTCGGTATGTAGCTTCTGAACTTGAAGATACTTTAGGTGG‑TGTCTACTCTATCCTTTCTCAAGAATTACAATTTCCTCTGGTACGAGTGCTCTTGAAGCAACTACAAGCCACGCAACAGATTCCTGAGTTACCTAAGGAAGCCGTagag                                                       <  1:315255/301‑1 (MQ=255)`  
                                                                                                                                                                                                                                              `tcCAGAAGACATCTCGTTCCTCCAACTGGTGAAGCAAGCAGACTTTCCTGTAGCTAACGCCGCAAGTGACGCTATCGCGGCTCGCCTTTCGTTAGCCTTTATGTTGAACTCTGCGGTTCAGCGTACAGGGGAACGTGTGACGGCCGATGAGATTCGGTCTGTAGCTTCTGCACTTGAAGATCCTCTAGGTGG‑TGTCTACTCTCTCCTTTCTCAAGAATTACAATTGCCTCTGGGTCGAGTGCCCCTGAAGCAACTACAATCCACGCAACAAATTCCTCAATTGCCTAAGGAATCCGTatag                                                       >  1:24422/1‑301 (MQ=255)`  
                                                                                                                                                                                                                                              `cccAGAAGACATCTAGTTCCTCCAACTGGAGAAGCAAGCAGACTTTACTGTAGCTAACGCCGTAAGTGACGCTATCGCGGCTCGCCTTTCGTTTGCCTTTATGTTGAACTCTGCGGTTCAGCGTACAGGTGAACGTGTGACCGCCGAAGAGATTCGGTATGTAGCTTCTGAACTTGAAGATACTTTAGGTGG‑TGTCTACTCTATCCTTTCTCAAGAATTACAATTGCCTCTGGTACGAGTGCTCTTGAAGCAACTACAAGCCACGCAACAGATTCCTGAGTTACCTAAGGAAGCCGTagag                                                       <  1:241429/300‑1 (MQ=255)`  
                                                                                                                                                                                                                                               `ccAGAAGACATCTCGTTCCTCCAACTGTAGAAGCAAGCAGACTTTACTGTAGCTAACGCCGTAAGTGACGCTATCGCGGCTCGCCTTTCGTTTGCCTTTATGTTGAACTCTGCGGTTCAGCGTACAGGTGAACGTGTGACCGCCGAAGAGATTCGGTATGTAGCTTCTGAACTTGAAGATACTTTAGGTGG‑TGTCTACTCTATCCTTTCTCAAGAATTACAATTGCCTCTGGTACGAGTGCTCTTGAAGCAACTACAAGCCACGCAACAGATTCCTGAGTTACCTAAGGAAGCCGTAGAGc                                                      <  1:54591/301‑1 (MQ=255)`  
                                                                                                                                                                                                                                               `ccAGAAGACATCTCGTTCCTCCAACTGGAGAAGCAAGCAGACTTTACTGTAGCTAACGCCGTAAGTGACGCTATCGCGGCTCGCCTTTCGTTTGCCTTTATGTTGAACTCTGCGGTTCAGCGTACAGGTGAACGTGTGACCGCCGAAGAGATTCGGTATGTAGCTACTGAACTTGAAGATACTTTAGGTGG‑TGTCTACTCTATCCTTTCTCAAGAATTACAATTGCCTCTGGTACGAGTGCTCTTGAAGCAACTACAAGCCACGCAACAGATTCCTGAGTTACCTAAGGAAGCCGTAGAGc                                                      <  1:173493/301‑1 (MQ=255)`  
                                                                                                                                                                                                                                                            `cGTTTCTCCAACTGGAGAAGCAAGCAGACTTTACTGTAGCTAACGCCGTAAGTGACGCTATCGCGGCTCGCCTTTCGTTTGCCTTTATGGTGAACTCTGCGGTTCAGCGTACAGGTGAACGGGTGACCGCCGAAGAGATTCGGTATGTAGCTTCTGAACTTGAAGATACTTTAGGTGG‑TGTCTACTCTATCCTTTCTCAATAATTACAATTGCCTCTGGTACGAGTGCTCTTGAAGCAACTACAAGCCACGCAACAGATTCCTGAGTTACCTAAGGAAGCCGTAGAGCCAACCATTAGTAc                                         <  1:86878/301‑1 (MQ=255)`  
                                                                                                                                                                                                                                                            `cGTTCCTCCAACTGGAGAAGCAAGCAGACTTTACTGTAGCTAACGCCGTAAGTGACGCTATCGAGGCTCGCCTTTCGTTTGCCTTTATGTTGAACTCTGCGGTTCAGCGTACAGGTGAACGTGTGACCGCCGAAGAGATTCGGTATGTAGCTTCTGAACTTGAAGATACTTTAGGTGG‑TGTCTACTCTATCCTTTCTCAAGAATTACAATTGCCTCTGGTACGAGTGCTCTTGAAGCAACTACAAGCCACGCAACAGATTCCTGAGTTACCTAAGGAAGCCGTAGAGCCAACCATTAGTAc                                         <  1:14955/301‑1 (MQ=255)`  
                                                                                                                                                                                                                                                               `tcctccAACTGTAGAAGCAAGCAGACTTTACTGTAGCTAACGCCGTAAGTGACGCTATCGCGGCTCGCCTTTCGTTTGCCTTTATGTTGAACTCTGCGGTTCAGCGTACAGGTGAACGTGTGACCGCCGAAGAGATTCGGTATGTAGCTTCTGAACTTGAAGATACTTTAGGTGG‑TGTCTACTCTATCCTTTCTCAAGAATTACAATTGCCTCTGGTACGAGTGCTCTTGAAGCAACTACAAGCCACGCAACAGATTCCTGAGTTACCTAAGGAAGCCGTAGAGCCAACCATTAGTACAgg                                      <  1:64685/301‑1 (MQ=255)`  
                                                                                                                                                                                                                                                                       `cTGGAGAAGCAAGCAGACTTTACTGTAGCTAACGCCGTAAGTGACGCTATCGCGGCTCGCCTGTCGTTTGCCTTTATGTTGAACTCGGCGGTTCAGCGTACAGGTGAACGTGTGACCGCCGAAGAGATTCGGTATGTAGCTTCTGAACTTGAAGATACTTTAGGTGG‑TGTCTACTCTATCCTTTCTCAAGAATTACAATTGCCTCTGGTACGAGTGCTCTTGAAGCAACTACAAGCCACGCAACAGATTCCTGAGTTACCTAAGGAAGCCGTAGAGCCAACCATTAGTACAGGTCTGGAAg                              <  1:101997/301‑1 (MQ=255)`  
                                                                                                                                                                                                                                                                           `ggaAGCAAGCAGACTTTACTGGAGCTAACGCCGTAAGTGACGCTATCGCGTCTCTCCTTTCTTCTGCCTTTATGTTGAACTCTGCGGTTCAGCGTACAGGTTAACGTGTGACCGCAGAAGAGATTCGGTATGTAGCTTCTGAACTTGAAGATACTTTAGGTGG‑TGTCTACTCTATCCTTTCTCAAGAATTCCCATTGCCTCTGGTACGAGTGCTCTTGAAGCAACTACAAGCCACGCAACAGATTCCTGAGTTACCTAAGGAAGCCGTAGAGCCAACCATTAGTACAGGTCTGGAAGCAAt                          <  1:331222/300‑1 (MQ=255)`  
                                                                                                                                                                                                                                                                              `agcaagcaGACTTTACTGTAGCTAACGCCGTAAGTGACGCTATCGCGGCTCGCCTTTCGTTTGCCTTTATGTTGAACTCTGCGGTTCAGCGTACAGGTGAACGTGTGACCGCCGAAGAGATTCGGTATGTAGCTTCTGAACTTGAAGATACTTTAGGTGG‑TGTCTACTCTATCCTTTCTCAAGAATTACAATTGCCTCTGGTACGAGTGCTCTTGAAGCAACTACAAGCCACGCAACAGATTCCTGAGTTACCTAAGGAAGCCGTAGAGCCAACCATTAGTACAGGTCTGGAAGCAATTgg                       >  1:297350/1‑301 (MQ=255)`  
                                                                                                                                                                                                                                                                               `gcaagcaGACTTTACTGTAGCTAACGCCGTAAGTGACGCTATCGAGGCTCGCCTTTCGTTTGCCTTTATGTTGAACTCTGCGGATCAGCGTACAGGTGAACGTGTGACCGCCGAAGTGATTCGGCATGTAGCTTCTGAACTTGAAGATACTTTAGGTGG‑TGTCTACTCTATCCTTTCTCAAGAATTACAATTGCATCTGGTACGAGTGCTCTTGAAGCAACTACAACCCACGCAACAGATTCCTGAGTTACCTATGTAAGCCGTAGAGCCAACCATTAGTACAGGTCTGGAAGCAATTGGt                      >  1:247588/1‑301 (MQ=255)`  
                                                                                                                                                                                                                                                                                 `aagcaGACTTTACTGTAGCTAACGCCGTAAGTGACGATATCGAGGCTCGCCTTTCGTTTGCCTTTATGTTGAACTCTGCGGTTCAGCGTACAGGTGAACGTGTGACCGCCGAAGAGATTCGGTATGTAGCTTCTGAACTTGAAGATACTTTAGGTGG‑TGTCTACTCTATCCTTTCTCAAGAATTACAATTGCCTCTGGTACGAGTGCTCTTGAAGCAACTACAAGCCACGCAACAGATTCCTGAGTTACCTAAGGAAGCCGTAGAGCCAACCATTAGTACAGGTCTGGAAGCAATTGGTCg                    <  1:96114/301‑1 (MQ=255)`  
                                                                                                                                                                                                                                                                                      `gACTTTACTGTAGCTAACGCCGTAAGTGACGCTATCGCGGCTCGCCTTTCGTTTGCCTTTATGTTGAACTCTGCGGTTCAGCGTACAGGTGAACGTGTGACCGCCGAAGAGATTCGGTATGTAGCTTCTGAACTTGAAGATACTTTAGGTGG‑TGTCTACTCTATCCTTTCTCAAGAATTACAATTGCCTCTGGTACGAGTGCTCTTGAAGCAACTACAAGCCACGCAACAGATTCCTGAGTTACCTAAGGAAGCCGTAGAGCCAACCATTCGTACAGGTCTGGAAGCAATTGGTCGAGGAc               >  1:342417/1‑301 (MQ=255)`  
                                                                                                                                                                                                                                                                                        `cTTTACTGTAGCTAACGCCGTAAGTGACGCTATCGCGGCTCGCCTTTCGTTTGCCTTTATGTTGAACTCTGCGGTTCAGCGTACAGGTGAACGTGTGACCGCCGAAGAGATTCGGTATGTAGCTTCTGAACTTGAAGATACTTTAGGTGG‑TGTCTACTCTATCCTTTCTCAAGAATTGCAATTGCCTCTGGTACGAGTGCTCTTGAAGCAACTACAAGCCACGCAACAGATTCCTGAGTTACCTAAGGAAGCCGTAGAGCCAACCATTAGTACCTGTCtcttatacccatctccgagccca             >  1:113115/1‑279 (MQ=255)`  
                                                                                                                                                                                                                                                                                                 `aGCTAACGCCGTAAGTGACGCTATCGCGGCTCGCCTTTCGTTTGCCTTTATGTTGAACTCTGCGGTTCAGCGTACAGGTGAACGTGTGACCGCCGAAGAGATTCGGTATGTAGCTTCTGAACTTGAAGATACTTTAGGTGG‑TGTCTACTCTATCCTTTCTCAAGAATTACAATTGCCTCTGGTACGAGTGCTCTTGAAGCAACTACAAGCCACGCAACAGATTCCTGAGTTACCTATGGAAGCCGTAGAGCCAACCATTAGTACAGTTCTGGAAGCAATTGGTCTAGGACAAGACCTTGAt    >  1:340594/1‑301 (MQ=255)`  
                                                                                                                                                                                                                                                                                                  `gagacaGCCGTAAGTGACGCTATCGCGGCTCGCCTTTCGTTTGCCTTTATGTTGAACTCTGCGGTTCAGCGTACAGGTGAACGTGTGACCGCCGAAGAGATTCGGTATGTAGCTTCTGAACTTGAAGATACTTTAGGTGG‑TGTCTACTCTATCCTTTCTCAAGAATTACAATTGCCTCTGGTACGAGTGCTCTTGAAGCAACTACAAGCCACGCAACAGATTCCTGAGTTACCTAAGGAAGCCGTAGAGCCAACCATTAGTACAGGTCTGGAAGCAATTGGTCGAGGACAAGACCTTGATa   <  1:261020/296‑1 (MQ=255)`  

                                                                                                                                                                                                                                                                                                       `|`                                                                                                                                                                                                                                                                                                           
`ATCCTAAAGAGGCTTGCCCATACATCCCGATTCGGATGGTCAGACTAGATGGTGAATCCTACGGTCGTTCGTACATTGAGGAATACTTAGGTGACTTACGGTCCCTTGAAAATCTCCAAGAGGCTATCGTCAAGATGTCCATGATTAGCTCTAAGGTTATCGGCTTAGTGAATCCTGCTGGTATCACCCAGCCACGCCGACTGACCAAAGCTCAGACTGGTGACTTCGTTACTGGTCGTCCAGAAGACATCTCGTTCCTCCAACTGGAGAAGCAAGCAGACTTTACTGTAGCTAAAGCCGTAAGTGACGCTATCGAGGCTCGCCTTTCGTTTGCCTTTATGTTGAACTCTGCGGTTCAGCGTACAGGTGAACGTGTGACCGCCGAAGAGATTCGGTATGTAGCTTCTGAACTTGAAGATACTTTAGGTGG‑TGTCTACTCTATCCTTTCTCAAGAATTACAATTGCCTCTGGTACGAGTGCTCTTGAAGCAACTACAAGCCACGCAACAGATTCCTGAGTTACCTAAGGAAGCCGTAGAGCCAACCATTAGTACAGGTCTGGAAGCAATTGGTCGAGGACAAGACCTTGATAA  >  NC_001604.1/20970‑21561`

  

Alignment Legend

Aligned base mismatch/match (shaded by quality score): `ATCG/ATCG` `< 3 ≤` `ATCG/ATCG` `< 11 ≤` `ATCG/ATCG` `< 24 ≤` `ATCG/ATCG` `< 35 ≤` `ATCG/ATCG` `< 38 ≤` `ATCG/ATCG`

Unaligned base: `atcg`    Masked matching base: `atcg`    Alignment gap: `‑`    Deleted base: `‑`
